# Impasse thérapeutique pour une tumeur de la vessie métastatique métachrone à un sarcome de kaposi: à propos d’un cas rare

**DOI:** 10.11604/pamj.2019.32.218.16007

**Published:** 2019-04-30

**Authors:** Bienvenu Bega Shamalirwa, Omana Wembonyama, Richepin Tidahy, Soufiane Mellas, Jalal Eddine El Ammari, Mohammed Fadl Tazi, Moulay Hassan Farih, Hakima Elmahi, Ibrahim Sory Sidibe

**Affiliations:** 1 Service d'Urologie, CHU Hassan II, Pharmacie de l'Université Sidi Mohamed Ben Abdellah, Fèz, Maroc; 2 Service de Dermatologie, CHU Hassan II, Pharmacie de l'Université Sidi Mohamed Ben Abdellah, Fèz, Maroc; 3 Laboratoire d'Analyses Anatomo-pathologique, CHU Hassan II de Fèz, Pharmacie de l'Université Sidi Mohammed Ben Abdellah, Fèz, Maroc

**Keywords:** Carcinome urothélial de la vessie métastatique, sarcome de kaposi, chimiothérapie, immunodepression, Metastatic urothelial bladder cancer, Kaposi sarcoma, chemotherapy, immunodepression

## Abstract

La chimiothérapie est le traitement indiqué pour un carcinome urothélial métastatique de la vessie, la coexistence d'une tumeur de vessie métastatique avec un sarcome de Kaposi pose un sérieux problème d'aggraver le nouveau néoplasie, un cas rare où n'avons pu que confier le patient à sa famille.

## Abstract (en)

Chemotherapy is the treatment of choice for a metastatic urothelial bladder cancer. We here report a rare case of metastatic bladder cancer associated with Kaposi sarcoma posing a serious risk of aggravation of the new neoplasia. It was necessary to entrust the patient to his family.

## Introduction

Le carcinome urothélial de la vessie, deuxième cancer urologique de l'homme de par sa prévalence, est caractérisé par évolution lente marquée par des récidives et la progression de la pathologie tumorale d'où l'importance d'une prise en charge adéquate. Lorsque le consentement éclairé du patient pose problème, on peut aboutir à une impasse thérapeutique.

## Patient et observation

**Figure 1:**
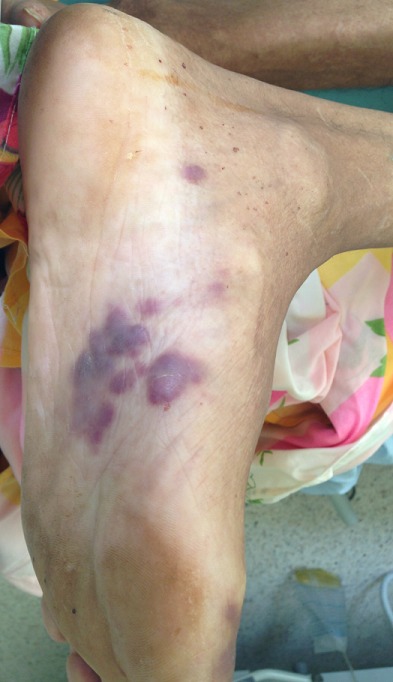
lésions cutanées des tâches hémorragiques suspectes au niveau de la plante des pieds (le pied gauche du patient)

**Figure 2:**
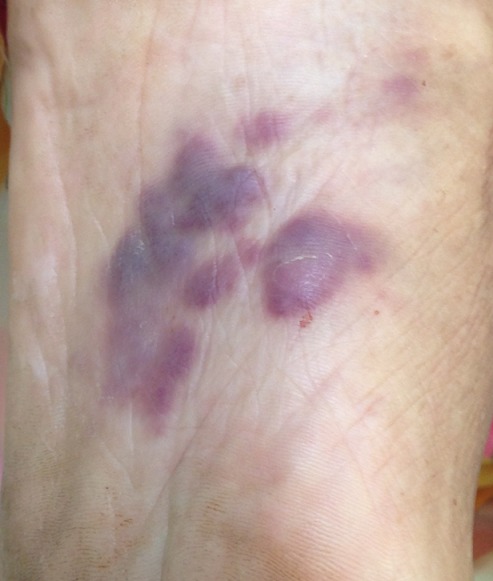
zoom sur les lésions cutanées de la plante du pied gauche

Nous vous rapportons le cas d'un patient de 72 ans, tabagique chronique pendant 10 ans sevré depuis 30 ans, chez qui le diagnostic de carcinome urothélial de la vessie n'infiltrant pas le muscle était posé depuis plus de 12 ans et avait déjà bénéficié de 4 résections trans uréthrale de la vessie puis dans l'évolution, le patient sera perdu de vue durant 3 ans (il ne s'était plus présenté au service pour les contrôles et par conséquent il n'avait pas bénéficié de l'immunothérapie) jusqu'à ce qu'il consulte aux urgences pour une rétention aigue d'urine sur caillots (hématurie caillotante compliquée d'une rétention aigue d'urine), l'échographie vésico-rénale réalisée va mettre en évidence une dilatation urétéro pyélocalicielle bilatérale modérée en amont d'une vessie pour laquelle le patient va bénéficier de la mise en place des néphrostomies bilatérales. On va constater aussi sur le patient; un état cachectique, l'apparition des tâches hémorragiques suspectes au niveau de la plante des pieds (Figure 1, Figure 2) pour lesquelles un avis dermatologique sera sollicité ainsi qu'un bilan d'extension pour actualisation de son état de santé.

**Figure 3:**
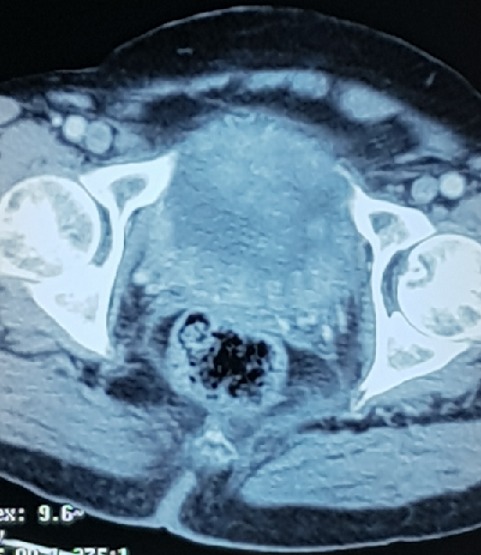
coupe transversale du scanner abdomino-pelvien montrant la tumeur de la vessie d’allure infiltrante

**Figure 4:**
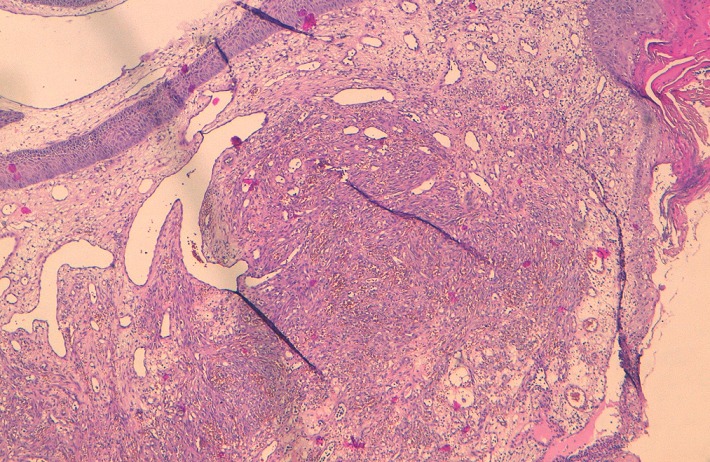
coupe histologique au grossissement X5 montrant l’épithélium malpighien hyperkératinisant parakératosique, on note un chorion fait des faisceaux entrecroisés, avec des fentes vasculaires courtes

**Figure 5:**
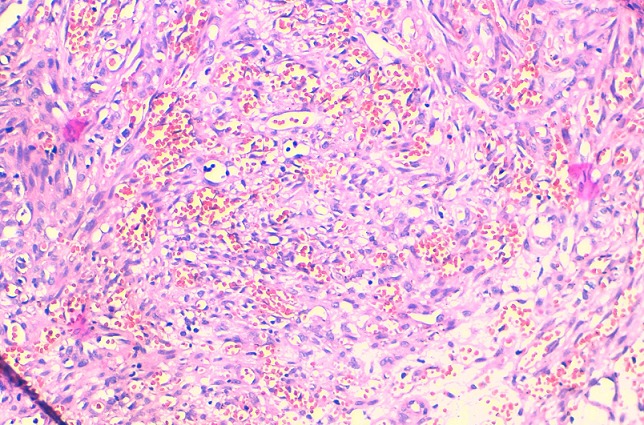
coupe histologique au grossissement X20 montrant des cellules fusiformes à orientation parallèle et à faisceaux entrecroisés dont une étude immuno-histochimique a été réalisée: les cellules tumorales expriment le CD34

**Figure 6:**
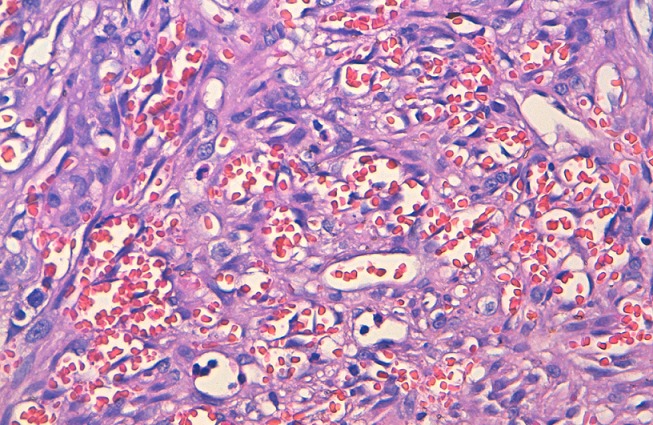
coupe histologique au grossissement X40 montrant des figures de mitoses, des hématies présentes dans la lumière des parois vasculaires, des cellules fusiformes à orientation parallèle et à faisceaux entrecroisés le ki est estimé à environ 10%

Le scanner thoraco abdomino-pelvien va conclure à: un processus tissulaire bourgeonnant, infiltrant toute la paroi vésicale (Figure 3) envahissant les méats urétéraux et déterminant une urétéro hydronéphrose modérée bilatérale, des poly adénopathies des 2 chaînes iliaques internes et externes, primitives et lombo aortiques, des lésions ostéolytiques de l'aile iliaque gauche et de l'hémi sacrum homolatéral ainsi que des multiples nodules et micronodules parenchymateux de deux champs pulmonaires évocateurs de lésions métastatiques. L'équipe dermatologique va procéder à une biopsie cutanée de ces lésions, le résultat anatomo pathologique va conclure en faveur d'un Sarcome de Kaposi (Figure 4, Figure 5, Figure 6). Sur le plan de sa pathologie urothélial, le patient sera adressé en oncologie médicale pour une chimiothérapie. Mais au cours des réunions des concertations pluri disciplinaires, il sera relevé que l'immunodépression induite par la chimiothérapie risque d'aggraver sensiblement le sarcome de kaposi constituant ainsi une impasse thérapeutique. La décision finale sera de confier le patient à sa famille, le patient va décéder 2 mois après sa sortie de l'hôpital.

## Discussion

La coexistence de plusieurs cancers primitifs chez un même individu est un phénomène connu dans la littérature oncologique avec une fréquence évaluée entre 2,6% et 3,9% tous cancers confondus [1, 2]. La survenue du sarcome de Kaposi convient bien d'être classée dans les tumeurs métachrones [3]. Le sarcome de Kaposi est une néoplasie rare des cellules endothéliales. Il est caractérisé par l'apparition de lésions violacées maculaires qui progressent pour devenir des papules et des plaques quelque fois en nodules et rarement des tumeurs. Le sarcome de Kaposi est décrit comme une pathologie rare survenant chez les immunodéprimés [4] l'immunodépression semble jouer un rôle déterminant au cours de certaines formes de la maladie de Kaposie (sida, transplantés), notre patient avait une séronégativité au VIH et n'a jamais été transplanté. Il n'existe cependant pas, de déficit immunitaire détectable avec nos moyens actuels au cours de la maladie de Kaposi classique. Une prédisposition génétique est possible, mais reste à préciser [5, 6].

Toutes fois des facteurs hormonaux [5, 6], l'appartenance au sexe masculin a été rapportée comme un facteur de risque pour développer un Sarcome de Kaposi [7] ajouté au fait que notre patient a été tabagique chronique pendant 10 ans en sachant qu'une étude réalisée par Geodert *et al.* a noté que le tabagisme est associé avec un taux statistiquement significatif à faible risque de développer un sarcome de kaposi [8]; tous facteurs réunis peuvent expliquer le sarcome de Kaposi chez notre patient. Notre patient était diagnostiqué d'un carcinome urothélial depuis 12 ans, les difficultés que ce patient nous a posé durant son suivi médical, ont permis à ce que sa pathologie puisse récidiver à 4 reprises puis de progresser en un carcinome urothéliale infiltrant le muscle avec des métastases à distance, son cas était devenu une indication de la chimiothérapie malheureusement l'immunodépression que cette chimiothérapie allait induire constituait un risque majeur d'aggraver le sarcome de Kaposi réalisant ainsi un cas de carcinome urothélial de la vessie métastatique avec une impasse thérapeutique.

## Conclusion

Le carcinome urothélial infiltrant le muscle vésical avec des métastases à distance constitue une indication de chimiothérapie qui va induire une immunodépression chez le patient mais si cette immunodépression devient un facteur de risque d'aggraver une pathologie comme le sarcome de Kaposi, on se retrouve bien devant une impasse thérapeutique.

## Conflits d’intérêts

Les auteurs ne déclarent aucun conflit d'intérêts.
